# Case of Fracture of the Occipital Bone with the Loss of a Portion of the Cerebellum, Terminating Favorably

**Published:** 1804-07-01

**Authors:** J. Evans

**Affiliations:** Ketley, Shropshire


### Case *of* Fracture *of the* Occipital Bone *with the* Loss *of a* Portion *of the* Cerebellum, *terminating favorably. By* J. Evans, *of Ketley, Shropshire.*

On the 2d of December, 1803, I was sent for to John Johnson, aged 27 years, servant to Joseph Reynolds, Esq. of this place, who half an hour before had received a kick upon the posterior part of his head, from a large waggon horse in his master's stable. On my arrival I discovered a wound in the scalp, about an inch and a half in length, filled with brain. Upon examination I could plainly feel a part of the skull considerably depressed near the attachment ment of the Trapezius muscle. The man had lost some ounces of blood on the spot where the accident happened, but the hæmorrhage had ceased when I saw him. I found him tolerably rational, but slow in answering questions; his pulse feeble and irregular. I directed a bread and milk poultice to be applied to the injured part, and an opiate to be given him in the evening, which his stomach rejected. He passed that night without much pain. The following morning the portion of brain which lay in the wound (being the size of a small walnut) came away with the poultice. Apprehending fatal consequences were likely to ensue from so severe an injury, I requested to have a consultation with Mr. Yonge, of Shiffnal, a gentleman highly respected for his professional abilities. We were unanimous in our opinion, that while the man remained free from symptoms of oppression, it would be most prudent to leave the business to nature, without attempting to elevate the bone. His bowels were kept in a proper state by small doses of castor oil, and the pain of his head (which was sometimes violent) was occasionally alleviated by opiates. Dry lint and a bread and milk poultice were the applications to the wound during the suppurative process; when the discharge became less, lint covered with a thin piece of sponge were the only dressings made use of. On the tenth day from the accident the patient had a considerable hæmorrhage from his nose, which seemed of service to him in relieving an excruciating pain in his head, which he at that time complained of. At the expiration of five weeks his usual faculties and spirits were quite restored. Previous to that period he said his sight had been very imperfect, although he never complained of it, and was extremely low from the danger he apprehended himself to be in. During his confinement, his diet consisted chiefly of milk, broth, and tea, according to his inclination. Being naturally a sober man, he would not suffer his attendants to give him any thing that was heating. On the 20th of February, he was well enough to return to his usual employ, but his wound was not healed of some weeks after. The depressed portion of the os occipitis was near the *third* of an inch below its natural level; being not wholly detached, no exfoliation took place. Is it not probable, that the portion of brain forced out of the skull made a sufficient space for the depressed bone to lodge in, without causing those alarming symptoms which generally attend such injuries? The depression is now so large as to admit the end of a moderate sized thumb.

*June* 12, 1801.

OBSERVATIONS

